# Hyperspectral Classification of Blood-Like Substances Using Machine Learning Methods Combined with Genetic Algorithms in Transductive and Inductive Scenarios

**DOI:** 10.3390/s21072293

**Published:** 2021-03-25

**Authors:** Filip Pałka, Wojciech Książek, Paweł Pławiak, Michał Romaszewski, Kamil Książek

**Affiliations:** 1 Department of Computer Science, Faculty of Computer Science and Telecommunications, Cracow University of Technology, 31-155 Krakow, Poland; filip.palka@pk.edu.pl (F.P.); wojciech.ksiazek@pk.edu.pl (W.K.); 2 Institute of Theoretical and Applied Informatics, Polish Academy of Sciences, 44-100 Gliwice, Poland; mromaszewski@iitis.pl (M.R.); kksiazek@iitis.pl or; 3 Department of Data Sciences and Engineering, Silesian University of Technology, 44-100 Gliwice, Poland

**Keywords:** hyperspectral classification, blood, SVM, genetic algorithm, machine learning

## Abstract

This study is focused on applying genetic algorithms (GAs) to model and band selection in hyperspectral image classification. We use a forensic-inspired data set of seven hyperspectral images with blood and five visually similar substances to test GA-optimised classifiers in two scenarios: when the training and test data come from the same image and when they come from different images, which is a more challenging task due to significant spectral differences. In our experiments, we compare GA with a classic model optimisation through a grid search. Our results show that GA-based model optimisation can reduce the number of bands and create an accurate classifier that outperforms the GS-based reference models, provided that, during model optimisation, it has access to examples similar to test data. We illustrate this with experiments highlighting the importance of a validation set.

## 1. Introduction

Genetic optimisation, inspired by natural evolution, is a well-known heuristic optimisation and search procedure that can be used for both feature and model selection in machine learning (ML). The focus of this paper is the use of genetic algorithms (GA) to train accurate ML algorithms; i.e., hyperspectral classifiers. A hyperspectral classifier aims to assign pixels in a hyperspectral image to predefined classes; e.g., different types of crops in an image of agricultural area. A hyperspectral pixel is a vector of measurements (typically, reflectance values) corresponding to a specific band: a narrow wavelength range of the electromagnetic spectrum. Since materials in the imaged scene uniquely reflect, absorb and emit electromagnetic radiation based on their molecular composition and texture, hyperspectral classification allows them to be accurately distinguished [1]. However, there are several challenges related to the task, such as the huge volume of images, their high dimensionality, the redundancy of information in hyperspectral bands and the presence of noise introduced by acquisition process and calibration procedures [2]. In addition, the observed spectra are mixtures (e.g., linear combinations) of material spectra in the imaged scene [3].

One particular challenge lies in the availability and quality of training data; i.e., the selection of a training set. Typically, due to the high cost of generating hyperspectral training examples [4], training sets in hyperspectral classification are small. However, when training pixels are randomly and uniformly sampled from the classified image itself, it is possible to achieve high accuracy even for very small training sets of 5–15 examples per class; e.g., by exploiting the spatial–spectral structure of the image and using semi-supervised learning [5]. This is because hyperspectral images provide highly distinctive features and because classes are usually relatively large in the image. In such problems, we may be more interested in finding the best assignment of pixels to classes than in finding the classification function itself. Therefore, referring to the concept of transductive learning proposed by Vapnik [6], we call such scenario a hyperspectral transductive classification (HTC) problem.

The challenge is elevated when training pixels come from a different image than test pixels. In such a case, differences in the acquisition environment (e.g., light intensity, time differences) and in-class spectra (e.g., different background materials in spectral mixtures) may be perceived as a complex noise. In such a scenario, the classifier is expected to generalise and compensate for the differences between the training set and classified data. In contrast to the HTC scenario, which treats the image as a “closed world”, we call this scenario a hyperspectral inductive classification (HIC), emphasising the importance of finding the best classification function. The HIC scenario shares similarities with the hyperspectral target detection problem [7], where spectra to be found in an image commonly come from spectral libraries.

Genetic algorithms [8,9] are well-established techniques for the selection of features and optimisation of classifier parameters. GAs are based on natural selection, inheritance and the evolutionary principle of the survival of the best-adapted individuals. Their advantages compared to the classic feature and model selection procedures such as grid search (GS) are, e.g., (a) their resistance to local extremes, (b) the ability to control selective pressure (exploration and exploitation) from global to local search and (c) ease of application due to feature selection being combined with parameter optimization. These advantages have resulted in GAs being frequently used for hyperspectral band selection [10] and the classification of multispectral [11] and hyperspectral data [12]. However, in most reference works, GAs are applied for a problem corresponding to the HTC scenario, typically using well-known hyperspectral datasets such as the “Indian Pines” or the “University of Pavia” images. Under such conditions, the simultaneous optimisation of classifier parameters with band selection allows researchers to achieve high classification accuracy [13]. To test both the HTC and HIC scenarios, in our experiments, we use a dataset described in [14] that consists of multiple hyperspectral images with blood and blood-like substances. The dataset is inspired by problems related to forensic analysis; e.g., the detection of blood. However, we focus on the problem of classification; i.e., distinguishing between classes corresponding to visually similar blood-like substances in the images. We use multiple images with the same classes but with significant spectral differences to compare the HTC and the HIC scenarios. We analyse the impact of GAs on the classification accuracy in comparison to the grid-search parameter selection using multiple state-of-the-art hyperspectral classifiers.

Our thesis is that hyperspectral classification with a GA-based model and band selection would allow more accurate classifiers to be obtained compared to the approach when parameters are selected with GS. To test this, we compare the accuracy of classifiers optimised with GA and GS in both the transductive and inductive hyperspectral classification scenarios. Our main contribution is the identification and experimental verification of the conditions under which GA outperforms GS in hyperspectral classification. We show that in order for this advantage to be significant, the classification problem must be sufficiently complex, such as in the HIC scenario, which is more difficult compared to the HTC. In addition, the data in the validation set used for model selection must be sufficiently similar to data in the test set, which is not always the case in the HIC. Since the use of GA can be time consuming compared to GS, our conclusions allow for a more informed choice of model selection method in various hyperspectral classification problems.

## 2. State of the Art

Machine learning algorithms are currently popular and widely used in medical imaging [15]. Some of the main areas of ML application are image segmentation—e.g., for melanin [16] or epidermis [17]—and segmentation and classification—e.g., for the detection of pigment network in dermoscopic images [18]. Due to the visibility of haemoglobin in the spectra, hyperspectral imaging (HSI) has become useful in areas related to medical diagnosis [19]. In addition, the detection and estimation of blood age in hyperspectral images [20] can be applied to forensic analysis [14]. However, the complexity of hyperspectral data makes the development of dedicated ML methods, especially classification algorithms, particularly important. Genetic algorithms are promising for the construction of hyperspectral classifiers as they enable simultaneous model selection and the reduction of data dimensionality.

### 2.1. Hyperspectral Classification

In this paper, we focus on spectral classification [1] which uses only spectral vectors. The leading approaches involve the use of Support Vector Machines (SVMs) [21], Extreme Learning Machines and their Kernel-based variants [22] or Multinomial Logistic Regression [23]. In order to further improve classification accuracy, spectral–spatial approaches [24] are employed. They make use of both pixel spectra and their spatial position in the image. In particular, a combination of spatial–spectral and semi-supervised approaches allows a high classification accuracy to be obtained, even for a small training set [5]. Recently, deep learning methods [25] are popular, although their limiting factor is the fact that they usually require relatively large training sets. However, some works, such as the approach presented in [26], based on residual networks, seem to be able to significantly reduce this dependency.

### 2.2. Evolutionary Computation and Genetic Algorithms

The advantages of techniques based on computational intelligence [27] methods lie in the properties inherited from their biological counterparts: the learning and generalization of knowledge (artificial neural networks [28]), global optimization (evolutionary computation [29]) and the use of imprecise terms (fuzzy systems [30]). The inspiration to undertake research on evolutionary computation (EC) [29] was the imitation of nature in its mechanism of natural selection, inheritance and functioning. Genetic algorithms (GAs) [31] are a part of evolutionary computation techniques, which have been used with success in fields such as the vehicle routing problem [32], feature selection [33], optimization [34], heart sound segmentation [35] or traveling salesman problem [36].

Genetic algorithms are one of the leading approaches to solve optimisation problems [9]. Due to the fact that they are computationally complex, they are often solved with heuristic methods, which make it possible to find a near-optimal solution faster. GA works by creating a population consisting of a selected number of individuals, each of them representing one solution to the problem. Then, from among all the individuals, those with the best results are selected and then subjected to genetic operators, which then create a new population. In particular, this technique can be applied for model selection to find parameters of a machine learning model and simultaneously perform feature selection, such as in works on heart arrhythmia detection [37,38], early diagnosis of hepatocellular cancer [39] or the prediction of credit scoring [40].

### 2.3. Hyperspectral Classification and Band Selection with GAs

GAs have been used many times for the classification and selection of characteristic wavelengths in hyperspectral data. For example, in [10], the authors use GA to find small subsets of the most distinctive bands. In [12], GAs are applied for band selection in preprocessed hyperspectral images in order to classify them. In [41], GA optimization is used to divide hyperspectral bands into three classes related to their discriminative power in the classification task. Authors verify their results using three standard hyperspectral datasets; i.e., the “University of Pavia”, “Indian Pines” and “Hekla”. The use of GAs for the simultaneous optimization of SVM parameters and band selection in HSI classification is presented in [13]. A similar scheme for multispectral data is used in [11], in which the authors emphasize the advantage of genetic algorithms over parameter optimization using a grid search. A very interesting use of a GA is presented in [42]: the authors apply a GA to a large number of hyperspectral cubes (111 images) in order to determine a subset of wavelengths characteristic for the identification of charcoal rot disease in soybean stems.

## 3. Materials and Methods

### 3.1. Dataset

We used the dataset described in [14], consisting of multiple hyperspectral images of blood and blood-like substances such as artificial blood, tomato concentrate or poster paint. Hyperspectral pixels in which these substances are visible were annotated by authors.

Images in the dataset were captured using a SOC710 hyperspectral camera operating in the spectral range of 377–1046 nm with 128 bands. Two types of images were used in our experiments: the “Frame” images, denoted as *F* in [14], which present classes on a uniform, white background; and the “Comparison” images (denoted as *E*), which present classes on diverse backgrounds consisting of multiple materials and fabrics.

**Figure 1:**
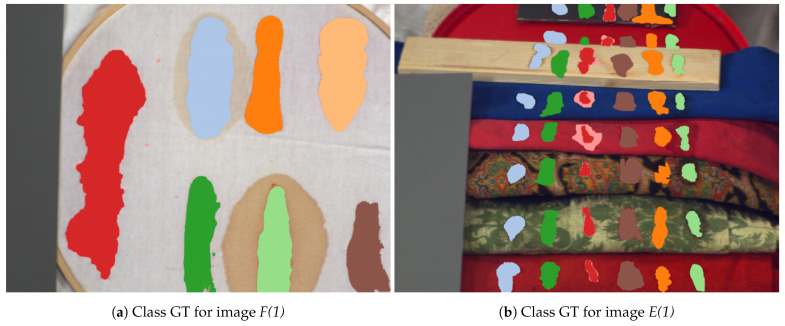

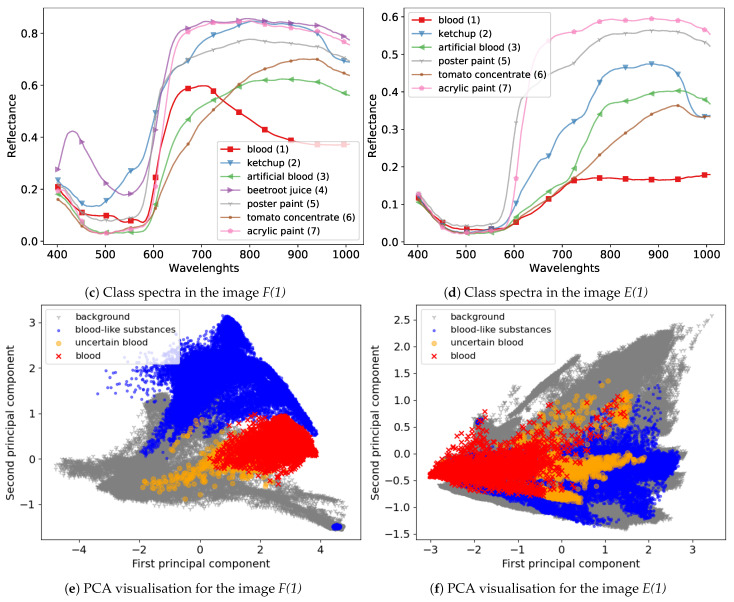
Visualisation of the dataset used in experiments. Upper panels present classes as a coloured ground truth on RGB images created from hyperspectral cubes. Middle panels present mean class spectra. Bottom panels present the PCA projection of data for the first two principal components. Images come from [14].

We used images captured on days {1,7,21}. Following the convention from [14], we denoted the day of acquisition after the scene name in brackets; e.g., *F(1)* for the scene “Frame” from day 1. The visualisation of the dataset is presented in Figure 1. Figure 1a,b presents the acquisition scenes for two selected images with marked pixels of different substances used during further experiments. Their mean spectra are presented in Figure 1c,d, while Figure 1e,f shows two components of the PCA projection. It is possible to observe that pixels marked in the *F(1)* image as “uncertain blood” have similar values of principal components to background pixels, while in the case of the *E(1)* image, “uncertain blood” is more similar to “blood-like substances”. Furthermore, spectra of different classes on the *F(1)* image are more diverse than in the case of the *E(1)* image, where pixels of various substances overlap according to the PCA projection.

### 3.2. Data Preprocessing

The aim of the initial preprocessing applied to dataset images was to reduce noise and compensate for uneven lighting. The following sequence of transformations was applied to every image:

1. Median filter: Images were smoothed with a spatial median filter with a window size of one pixel. This operation was intended to reduce the noise in spectra, using the fact that classes were spatially significantly larger than a single pixel.
2. Spectra normalization: As suggested in [14], the spectrum of each pixel was divided by its median. The purpose of this normalisation was to compensate for uneven lighting in the image.
3. Removal of noisy bands: Following [14], noisy bands (0–4), (48–50) and (122–128) were removed, leaving 113 bands.

### 3.3. Feature Extraction

In our experiments, we used derivative transformation to highlight important features of spectra. Derivative analysis [43] is a well-known method for transforming spectral signatures. Derivatives are sensitive to the shape of spectra; therefore, they are particularly effective in differentiating signals with characteristic spectral responses, such as haemoglobin response in blood [44], visible as peaks in wavelengths ∼542 nm and ∼576 nm (called α and β bands). We used first-order derivatives, computed as the difference between neighbouring bands.

**Figure 2:**
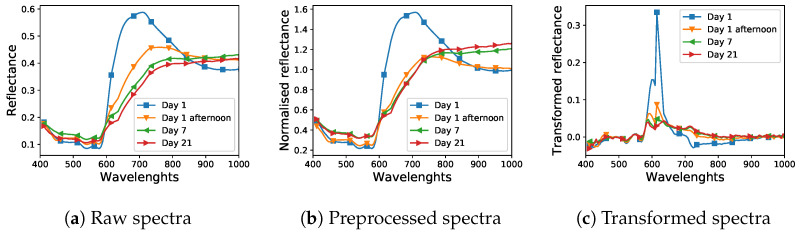
Visualisation of the impact of preprocessing and feature extraction on example spectra of the “blood” class from the dataset. Spectra in plot (**a**,**b**) were normalised by dividing each pixel by its median. Spectra in plot (**c**) were transformed by computing their first order derivatives.

A visualisation of the impact of preprocessing and feature extraction on example spectra is presented in Figure 2. Figure 2a,b presents the reflectance spectra of blood for different days after spilling, without and with the division of each pixel by its median value, respectively, while Figure 2c shows spectra after calculating first-order derivatives.

### 3.4. Classification Algorithms

#### 3.4.1. Support Vector Machines

In this work, we focus on the Support Vector Machine [45] (SVM) classifier, which is accurate in hyperspectral classification problems [1], including the classification of hyperspectral forensic data [46] and is well suited for optimisation with GA [13]. HSI classification with SVM can be described as follows:

Given a training set of labelled examples

(1) $$T=\left\{(x_{i},y_{i}),i=1,\ldots ,n\right\}\;x_{i}\in X\;y_{i}\in Y,$$

where X denotes a set of examples (e.g., hyperspectral pixels) and Y={−1,1} denotes the set of labels, the SVM classifies a hyperspectral example $x\in X\subset R^{d}$ using a function:

(2) $$f\left(x\right)=\mathrm{sgn}\left(\sum_{i=1}^{n}y_{i}\beta_{i}K\left(x,x_{i}\right)+b\right),$$

where $\beta_{i}\geq 0$ and *b* are coefficients computed through Lagrangian optimisation (margin maximisation on the training set). The kernel function K:X×X→R is used to compute the similarity measure between the classified example x and every training instance $x_{i}$.

We use three kernel functions:

- Gaussian radial basis function (RBF) $K\left(x_{i},x_{j}\right)=\exp(-\gamma ||x_{i}-x_{j}{\left|\right|}^{2})$, parameterised with {γ},
- sigmoid kernel $K\left(x_{i},x_{j}\right)=\tanh\left(\gamma x_{i}^{T}x_{j}+c_{0}\right)$ parametrised with $\left\{\gamma ,c_{0}\right\}$
- polynomial kernel $K\left(x_{i},x_{j}\right)={\left(\gamma x_{i}^{T}x_{j}+c_{0}\right)}^{d}$ parametrised with $\left\{\gamma ,c_{0},d\right\}$ that can be simplified to the linear kernel $K\left(x_{i},x_{j}\right)=x_{i}^{T}x_{j}$ when parameters $d=c_{0}=0$.

In addition to parameters of a chosen kernel, the SVM has an additional regularisation parameter, *C*, that controls the balance between the maximisation of the margin between classes and missclassification of examples. The value of this parameter must be fitted to a given problem, typically through cross-validation. However, the use of GA for selecting parameters is complicated by the fact that the value of *C* is unbounded from the above. Therefore, in our experiments, we used the classifier proposed in [47], namely the ν-SVM, which uses a bounded regularisation parameter ν∈(0,1〉, which is an upper bound on the fraction of misclassified examples from the training set and a lower bound on the fraction of support vectors.

#### 3.4.2. K-Nearest Neighbour (KNN)

The K-nearest neighbour algorithm (KNN) [48] belongs to the family of non-parametric models. The principle of operation of the algorithm is based on making predictions based on the closest neighbourhood of an example. A new, unclassified sample is labelled through a majority vote of a neighbourhood of a fixed size weighted by the distance of this sample from the voting neighbors. In our experiments, we used the Euclidean, the Manhattan and the Chebyshev distance measures.

#### 3.4.3. Multilayer Perceptron

A Multilayer Perceptron (MLP) [49] is a neural network composed of a combination of individual perceptrons that together form a multilayer structure. The most frequently distinguished layers are the input, hidden and output layer. Each layer may have a different number of neurons. Advanced network models consist of multiple hidden layers. The MLP is typically trained using a backpropagation algorithm. Despite its simplicity, the MLP achieves high accuracy on hyperpsectral data and is often used as a reference method for other algorithms [1].

### 3.5. Model and Feature Selection with Genetic Algorithms

We used genetic optimisation [9] to simultaneously select parameters of a machine learning model and perform feature selection. The ν-SVM [45] classifier was chosen for this type of optimisation due to its bounded parameterisation of the margin (see Section 3.4).

Taking advantage of the capabilities of the GA, which allow for the optimization of many parameters at once, in our implementation, the type of kernel function, kernel parameters, the regularization parameter and feature (hyperspectral band) selection were performed simultaneously. Table 1 presents the structure of a single individual. In our implementation, this individual consisted of one chromosome. The chromosome consisted of five genes responsible for the kernel type and its parameters and 113 genes responsible for hyperspectral bands.

**Figure 3:**
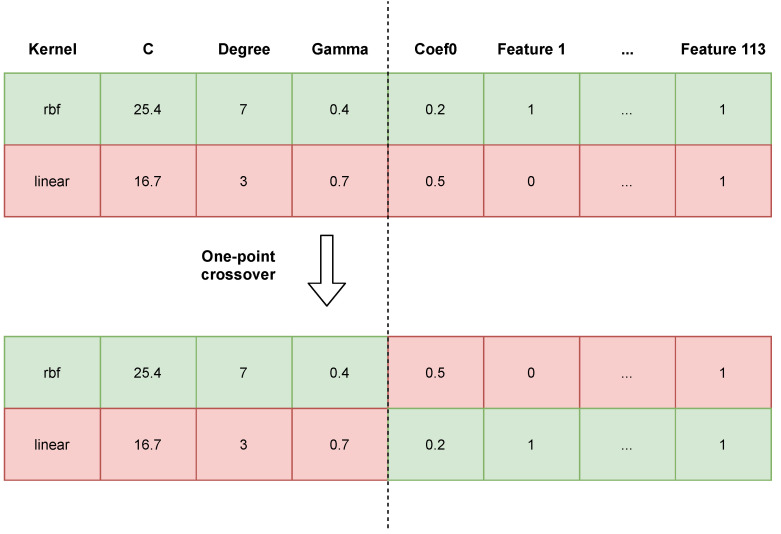
Visualisation of a one-point crossover between two individuals.

Figure 3 shows an example crossover between two individuals (i.e., classifiers). We observed that high probabilities of crossing and mutation had a positive effect on the search space; i.e., they allowed the search space to be better explored and for more solutions to be checked, reducing the chances of finding a locally optimal solution [50]. Thanks to the elitist strategy, there is a certainty that the best individual found will not be lost. The mutation of an individual consists in the modification of a single gene in the chromosome. If it is a gene responsible for a parameter of the SVM, its value is replaced by the new value of the given parameter from the set range (acceptable values are shown in Table 1). If we draw a gene that represents a feature, its value is replaced by the opposite one; e.g., from “not selected” (0) to “selected” (1). Values of our genetic algorithm parameters are presented in Table 2.

#### 3.5.1. Model Selection with Grid Search

In our experiments, grid search (GS) was used as a reference method for model selection. In many works, the SVM with the regularisation parameter *C* (denoted SVC) with an RBF kernel function has been used as a reference algorithm; therefore, we used this approach in addition to the ν-SVM. We also tested the KNN and MLP classifiers, as described in Section 3.4. Parameters of model selection with the GS are provided in Table 3.

#### 3.5.2. Implementation

All experiments were implemented in Python using the scikit-learn [52], PyTorch [53] and DEAP [54] libraries.

#### 3.5.3. Model Performance Metric

Because the number of examples in the classes of our dataset was similar, we used the accuracy as a performance metric, defined as follows:

(3) $$accuracy=\frac{1}{N}\left(\sum_{i=1}^{N}\frac{TP_{i}+TN_{i}}{TP_{i}+FP_{i}+TN_{i}+FN_{i}}\right)\times 100\%,$$

where *N* is the number of folds in cross validation, $TP_{i}$ denotes true positives, $TN_{i}$ denotes true negatives, $FP_{i}$ denotes false positives and $FN_{i}$ denotes false negatives.

## 4. Experiments

The main idea behind our experiments was to perform model and feature selection with GA and compare these results with a diverse set of classifiers trained classically; i.e., with a grid-search. Referring to classification scenarios introduced in Section 1, we considered three experimental scenarios:

1. Hyperspectral transductive classification (HTC)—training and test examples were randomly, uniformly selected from a single hyperspectral image.
2. Hyperspectral inductive classification (HIC)—training and test examples were selected from different images. Typically, training examples came from “Frame” images and testing examples came from the “Comparison” images.
3. Hyperspectral inductive classification with a validation Set (HICVS)—this scenario was similar to the HIC scenario: training examples came from “Frame” images and testing examples came from the “Comparison” images. However, model selection was performed using a separate validation set that was randomly, uniformly sampled from the “Comparison” scene. This scenario was designed to test the capabilities of GA optimisation under different conditions to those in the HIC scenario, which is discussed in detail in Section 6.

### 4.1. The Scheme of Experiments

The experiments can be divided into six stages:

1. Raw data—The data set consisted of seven hyperspectral images from the data set described in Section 3.1. Every image had 128 hyperspectral bands. The images represented two scenes—the “Frame” scene and the “Comparison” scene. Four of the seven images showed the “Frame” scene, captured in days $\left\{1,1_{a},7,21\right\}$, where the value $1_{a}$ represents the afternoon of the first day. The three “Comparison” images were captured on days {1,7,21}.
2. Data preprocessing—Data were transformed in accordance with the methodology described in Section 3.2: in order to reduce the effect of noise and uneven lighting, spectra were smoothed with the median window, normalised and noisy bands were removed. Background (unannotated pixels) and pixels from the class “beetroot juice” (class 4) that was not present in all images were removed. Finally, the problem was posed as a six-class classification with classes Y={1,2,3,5,6,7}.
3. Feature extraction—A derivative transformation was used, as described in Section 3.3.
4. Data split—Data were divided into training and test sets. A detailed description of this stage is included in Section 4.2, Section 4.3, Section 4.4.
5. Model optimization—Model and feature selection were performed as described in detail in Section 3.5. The reference method used for comparison was a grid search. In both cases, the accuracy was chosen as the evaluation criterion. The settings and details of the cross-validation varied depending on the scenario of the experiment; detailed descriptions are provided in descriptions of the individual scenarios.
6. Model evaluation—The final final results were expressed in terms of classification accuracy. After finding the best model in stage 5, this model was trained on the entire training set and tested on the test sets. The test sets were created from both scenes: “Frame” and “Comparison”. The training and testing process was repeated five times and the average accuracy with the standard deviation was calculated.

**Figure 4:**
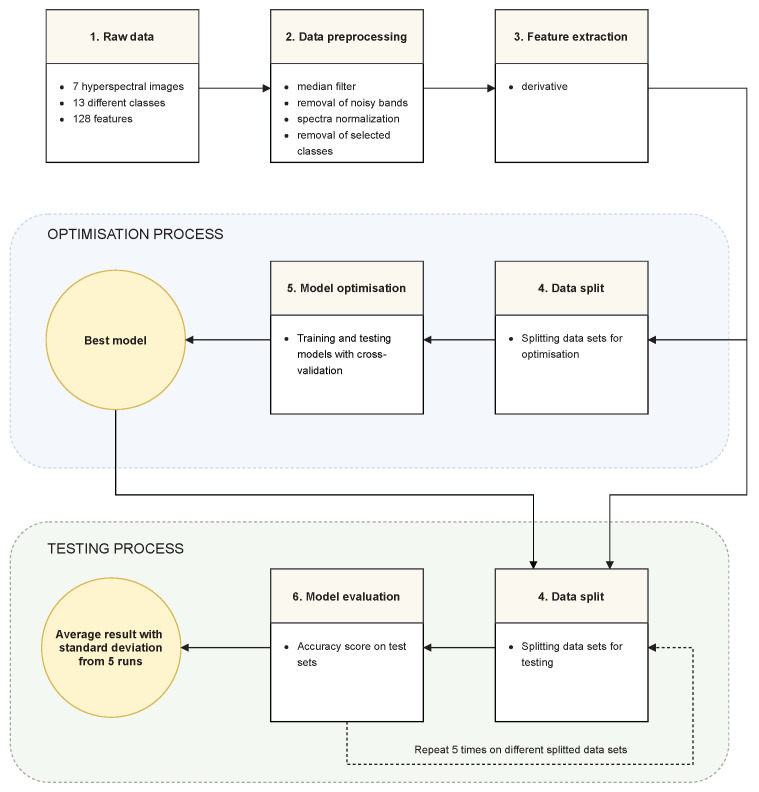
The overview scheme of experiments.

An overview schema of our experiments based on the above steps is presented in Figure 4. Transitions between successive stages are also described with a short summary of consecutive experiments phases.

### 4.2. Hyperspectral Transductive Classification (HTC)

In the HTC scenario training, pixels were randomly, uniformly sampled from the same images as test pixels. This scenario bore resemblance to a common hyperspectral classification setting, when classifiers are tested, e.g., using the “Indian Pines” data set [1]. The aim of this experiment was to test the capability of classifiers to model classes and distinguish between them.

The training set was a combination of examples from all images; i.e., “Frame” and “Comparison” scenes from all days. The training set consisted of an equal number of examples from each class and each day. We used the size of the least numerous class among all the images (989); therefore, the training set consisted of 41,538 hyperspectral pixels (989 pixels * six classes * seven images).

After selecting the best parameters and features using cross-validation on the training set, classifiers were trained on the whole training set and tested on the remaining examples.

### 4.3. Hyperspectral Inductive Classification (HIC)

In the HIC scenario, classifiers were trained on “Frame” images and tested on “Comparison” images. This scenario simulated a potential forensic application, where the model was prepared using laboratory samples and applied in the field in an unknown environment.

The training set size was 6000 examples (250 examples from each class, from four available images). The test set consisted of a total of 82,097 examples from “Comparison” scenes.

**Figure 5:**
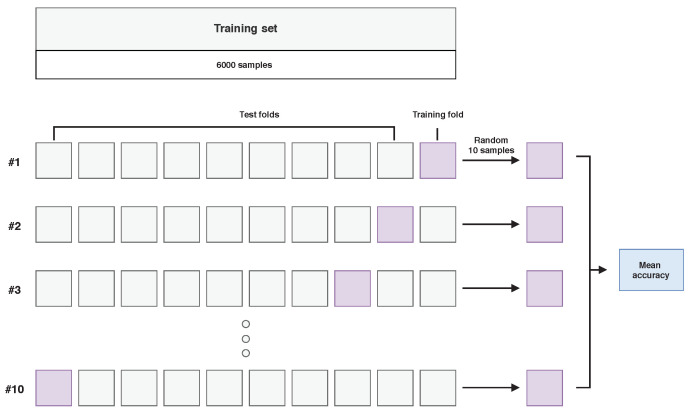
Visualisation of the model optimisation stage in the hyperspectral inductive classification (HIC) scenario, using 10-fold cross-validation on a selected training set from “Frame” images.

Each model was optimized in the process of a 10-fold cross-validation as visualised in Figure 5. Each time, one fold was used for training and the remaining ones for testing. Additionally, only a subset of 10 randomly selected examples from each class in the training set were used for training in a single cross-validation iteration. After the optimization stage, the best model was trained on examples in the training set and tested on the test set.

### 4.4. Hyperspectral Inductive Classification with a Validation Set (HICVS)

In the HICVS scenario, classifiers were trained on “Frame” images and tested on “Comparison” images, but in the model optimisation stage, a separate validation set was used, consisting of a subset of randomly, uniformly sampled examples from the “Comparison” images. The aim of this experiment was to determine and discuss the impact of applying GA in the model optimisation stage. The purpose was to test a scenario in which GA could perform the selection of features while maintaining model overfitting control. A discussion of this scenario is presented in the Section 6.

In the experiment, all examples from tests scenes from all days were divided into a test and a validation set in a ratio of 80% to 20%. Similarly to the HIC scenario, pixels from test scenes were not used as training examples. However, during the model optimization stage, models were tested on the validation set. The test set consisted of 65,676 examples and the validation set consisted of 16,421 examples.

**Figure 6:**
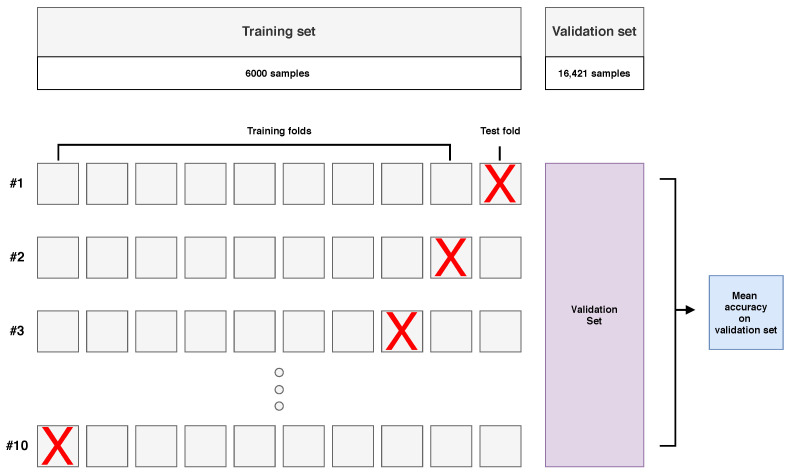
Visualisation of the model optimisation stage in the hyperspectral inductive classification with a validation set (HICVS) experiment with a small training set and 10-fold cross-validation.

The training set contained 6000 examples (250 examples from each class, from four available images). Models were trained using 10-fold cross-validation, as presented in Figure 6. Nine folds formed a training subset, and the model was tested on a validation set. The remaining fold was not involved in the validation process.

After the optimisation process, the best model was tested on a test set that did not contain examples from the validation set.

## 5. Results

This section presents our results divided into the three scenarios corresponding to experiments described in Section 4.

### 5.1. The HTC Scenario

The accuracies of all tested models on “Frame” images in the HTC scenario were close to 100%. Results for the “Comparison” images are presented in Table 4. The accuracy of all classifiers was the highest among the three tested scenarios (HTC, HIC and HICVS). Only the KNN classifier did not achieve an accuracy higher than 90%. The model based on the MLP classifier optimized with GS outperformed other classifiers in every case.

Interestingly, the accuracy for the image from the seventh day was higher than for the remaining images. This may result from time-induced changes in spectra, in particular from the oxidation of haemoglobin in the blood. On the first day, the spectra undergo significant changes, which may translate into high data variance and lower class cohesion. After a few days, the spectra (especially the blood) become more uniform, as can be seen in Figure 2a. Lower accuracy for significantly aged data after 21 days may result from the equalisation of spectral responses between classes as well as additional noise resulting, for example, from the presence of deposited dust.

### 5.2. The HIC Scenario

Results of the HIC scenario are presented in Table 5. The classifier trained with GA outperformed reference methods only on the first day, and even then, the ranges of standard deviations overlapped. For the remaining days, the SVM with a linear kernel scored best. Interestingly, the best kernel chosen by GA optimisation was also the linear kernel, and the number of bands was reduced from 113 to 61. We noticed that the training accuracy—i.e., the accuracy measured on the training set during model optimisation—was close to 100% for almost all models including the classifier trained with GA, which is consistent with the results of the HTC experiment.

### 5.3. HIC Scenario with a Validation Set

Results of the HICVS scenario experiments are presented in Table 6. In this scenario, the accuracy of almost all classifiers improved compared to the HIC scenario (see Table 5), but the GA-optimised classifier outperformed other methods. However, we also noticed an almost fourfold increase in standard deviation for the GA optimised model. Once again, the linear kernel was the winning model for GA and the number of selected bands was 64. Similarly to the HIC scenario, the training accuracy—i.e., the accuracy measured on the training set during model optimisation—was close to 100% for almost all models including the classifier trained with GA.

### 5.4. Computation Time

The computation time depended on the size of the training set and the number of folds in cross-validation. The computation in the HIC scenario took the least amount of time. Optimisation with GA took 13 min and that with GS 15 min. Optimisation calculations in the HICVS scenario took 2 h and 17 min for GA and 9 h and 54 min for GS. Optimisation in the HTC scenario took the longest time, with GA optimisation taking 7 h 28 min and GS optimisation taking 18 h and 12 min. Regarding the reference MLP architecture, the average training time was about 92.4 s in the case of HTC experiments and about 93.6 s in the case of the HIC scenario.

## 6. Discussion

### 6.1. The Impact of Preprocessing

The preprocessing described in Section 3.3 was done with the aim of extracting class features that were similar in all images. In order to illustrate the impact of the proposed preprocessing and data transformation on classification accuracy in the HTC and HIC scenarios, we performed a simple experiment: we repeated the HIC scenario; i.e., we trained the ν-SVM classifier obtained in the optimization process during the HIC scenario (including feature selection) with examples from all “Frame” images. However, we omitted step 3, “feature extraction”, from the procedure described in Section 4; i.e., the classifier processed normalised spectra. The training set size was 6000 examples (250 examples from each class, from four available images). The accuracy for the combined “Comparison” images was $acc_{\mathrm{Comp}.}^{\prime }=54.02\pm 0.21$, which was lower than the corresponding value in the Table 5; i.e., $acc_{\mathrm{Comp}}=66.54\pm 0.45$. At the same time, the accuracy for the remaining pixels of “Frame” images was $acc_{\mathrm{Frame}}^{\prime }=99.61\pm 0.05$, which was similar to the results of HTC experiments.

We conclude that, in the HTC scenario, where training and testing examples came from the same scene, the classifier was able to model classes and reach high classification accuracy even without preprocessing. However, the proposed preprocessing improved the accuracy in the HIC scenario, when the training and test were are more different.

### 6.2. Model Optimisation with GA in Hyperspectral Classification

Reference works on hyperspectral GA-based classification described in Section 2 present their advantages such as the reduction in data dimensionality through band selection, their resistance to overfitting or their consistently higher accuracy than for the reference model selected with GS [11]. However, most of the works consider only the HTC scenario, use similar, airplane or satellite-based images and sometimes compare the method with a model trained with preset parameters [13]. Therefore, to better assess the capability of GA-based model selection, we compared GA and GS in two scenarios that differed in regards to the complexity of the classification problem.

Our results show that in the HTC scenario, both model optimisation techniques resulted in comparable, highly accurate models. We noticed that the accuracy measured on the training set during the process of model optimisation was very similar to the final accuracy on the test set. It seems that for training and test sets created by randomly, uniformly sampling a hyperspectral image, spectra in both sets are similar enough that GA and GS-based model are comparable in regards to their accuracy, and the major advantage of GA in this scenario is the band selection, which more than halved the number of features in our experiments.

Compared to the HTC, the HIC scenario proved to be significantly more challenging. The accuracy values in Table 5 are lower compared to values in Table 4, and it seems that the GA-trained classifier was only slightly better than GS for images captured on the first day and scored second for test images captured on other days (although the number of features was once again halved). In the HIC scenario, training and test data came from images that differed in regards to the lightning conditions, spectral mixtures of imaged classes and the image background. We hypothesise that, despite the fact that both images contained the same, precisely applied and clearly visible substances, differences between the training and the test set were so significant that the selected model was overfitted. This is supported by the fact that, similar to the HTC scenario, the accuracy measured on the training set during the process of model optimisation was very high in the HIC. While GAs allow local maxima to be avoided during model optimization, when all training data are noisy in the same way, there is no global maximum that a GA could find. This hypothesis is further supported by the higher accuracy of the method on the first-day images. Images acquired on the first day were more similar since aging had a significant impact on spectra; e.g., the “blood” class spectrum changed significantly [44] due to haemoglobin oxidation.

In order to better explore the capabilities of GA in HSI model optimisation, we proposed one more experiment: the HICVS scenario described in detail in Section 4.4. In HICVS, the classifier was trained on a similar training set as in the HIC scenario, but during the model optimisation stage, the optimisation algorithm had access to examples in the validation set that were similar to test data. We expect that in this situation GA should gain an observable advantage over GS: since the algorithm can now control model overfitting through every epoch, it should be able to create a better generalizing classifier. Results in Table 6 confirm this hypothesis: while the results of the GS also improved, the improvement for GA was higher, and it scored first for all images.

Referring to our initial hypothesis introduced in Section 1 that GAs allow more accurate hyperspectral classifiers to bed obtained than GS, in our opinion, the presented results support this hypothesis, provided that certain assumptions related to the nature of the processed hyperspectral images are met. First, for a uniform data set, e.g., in the HTC scenario, when the training set is sufficient and uniformly sampled, both model optimisation methods can result in highly accurate, comparable classifiers. However, when spectra become noisy, which results in differences between the training and test sets, GA can outperform GS and avoid model overfitting, provided that a subset of examples similar to test data are available during model optimisation. When the noise between training and test data becomes too big, the advantage of GA over GS in terms of accuracy seems not significant. However, compared to GS, in all scenarios, GA can produce similar or more accurate classifiers while at the same time significantly reducing the dimensionality of the data through band selection.

## 7. Conclusions and Future Works

We compared a GA-based model selection with the classic approach based on a grid search in three different hyperspectral classification scenarios. In the hyperspectral transductive classification (HTC) scenario, the training and test data were taken from a single image, so they were similar. For this scenario, if a sufficiently large training set was available, both methods of model selection achieved comparable, very high accuracy. In the hyperspectral inductive classification (HIC) scenario, the training and test data came from different images, which negatively affected the accuracy of all tested classifiers. In this scenario, GAs only gained an advantage over GS for some images; e.g., day 1 image, where the characteristic blood features associated with haemoglobin spectral response were most visible. The third scenario, i.e., the hyperspectral inductive classification with a validation set (HICVS), was created on the basis of the HIC scenario. In the HICVS scenario, the model selection algorithm had access to examples similar to those in the test set, which allowed the GA-based optimisation to outperform GS for all images.

Our results show that for noisy data, as in HIC, the advantage of GA over GS in terms of accuracy is not significant and that in order to achieve this advantage, GA must have examples representative of the test set at the model selection stage; e.g., in the HICVS scenario. On the other hand, for a typical HTC scenario, existing approaches such as [5] or [25] allow very high accuracy to be obtained without an extensive search of the parameter space. This suggests that GA is a promising solution to challenging problems of hyperspectral classification, but its effective use imposes certain requirements on the available training data. This problem shares similarities with the problem of domain adaptation, described, e.g., in [55]. However, in all tested scenarios, the GA was able to generate models that were similar to or more accurate than GS while reducing the number of spectral bands by almost half.

We plan to apply the GA-based approach to different models, in particular recurrent neural networks, deep neural networks and ensemble learning. We would also like to test different feature extraction methods dedicated to the GA-based classification of hyperspectral images, especially in the HIC scenarios.

## Figures and Tables

**Table 1:** The structure of a chromosome corresponding to optimized parameters of the nu-SVM classifier along with selected hyperspectral bands. RBF: radial basis function.

Parameter	Range of Values
*K ^a^*	{RBF, polynomial, sigmoid}
ν	〈0.001,0.4〉
*d ^b^*	〈1,5〉
γ ^*c*^	〈0.001,5〉
$c_{0}$ *^d^*	〈0.01,10〉
band 1…113	{selected,notselected}

*^a^* Kernel function; *^b^* parameter of the polynomial kernel; *^c^* parameter of the RBF kernel; *^d^* parameter of the polynomial and sigmoid kernel.

**Table 2:** Parameters of the genetic algorithm (GA) used in experiments.

Parameter	Value
Size of the population	200
Number of epochs	100
Fitness function	Accuracy
Selection algorithm	Tournament selection, size 3
Crossover method	Uniform crossover
Mutation method	One-point mutation *^a^*
Probability of crossover	0.8
Probability of mutation	0.8
Elitist strategy	1 individual

*^a^* Own implementation.

**Table 3:** Grid-search (GS) parameters used in experiments.

Classifier	Parameter	Values
SVM	*K* *^a^*	{RBF, polynomial, sigmoid}
	*C*	〈0.001,1000〉
	*d* *^b^*	〈1,5〉
	γ *^c^*	〈0.001,5〉
	$c_{0}$ *^d^*	〈0.01,10〉
LSVM *^e^*	loss	{hinge, squared}
	*C*	〈0.001,1000〉
ν-SVM	*K* *^a^*	{RBF, polynomial, sigmoid}
	ν	〈0.001,0.4〉
	*d* *^b^*	〈1,5〉
	γ *^c^*	〈0.001,5〉
	$c_{0}$ *^d^*	〈0.01,10〉
KNN	Dist. metric	{Euclidean, Manhattan, Chebyshev}
	Weights	{Uniform, distance}
	No. neighbors	〈1,20〉
MLP	No. hidden layers	〈1,3〉
	Number of neurons on consecutive layers	$\left\{\{1000\},\{30,30\},\{1000,1000\},\{1000,1000,1000\}\right\}$
	Dropout	{0,0.5}
	Learning rate	{0.1,0.01,0.001}
	Batch size	{50,100}
	Number of iterations	{50,100,150,…,500}
	Weights initialisation	Glorot method [51] with normal distribution

^*a*^ Kernel function; ^*b*^ parameter of the polynomial kernel; ^*c*^ parameter of the RBF kernel; ^*d*^ parameter of the polynomial and sigmoid kernel; ^*e*^ linear Support Vector Machine (SVM), implemented in *liblinear* library. KNN: K-nearest neighbour; MLP: Multilayer Perceptron.

**Table 4:** Results of the HTC scenario for classification with GA and reference classifiers trained with a grid search (GS). The highest result in each day is denoted with a bold font. SVC: SVM with the regularisation parameter *C*.

Model Optimisation	Classifier	Accuracy/Day			
		1	7	21	All ^*b*^
GS	SVC	97.65±0.09	98.2±0.07	95.66±0.13	97.14±0.04
	LSVC *^a^*	91.19±0.63	91.25±0.54	87.89±0.44	90.13±0.52
	nu-SVM	97.02±0.1	97.87±0.12	95.27±0.15	96.66±0.07
	KNN	87.96±0.17	90.3±0.2	85.22±0.21	87.65±0.12
	MLP	98.94±0.10	99.32±0.06	98.31±0.12	98.83±0.07
GA	nu-SVM	98.05±0.11	98.51±0.07	96.48±0.11	97.66±0.05

^*a*^ SVM with a linear kernel; ^*b*^ results for combined data from all days.

**Table 5:** Results of the HIC scenario for classification with GA and reference classifiers trained with a grid search (GS). The highest result in each day is denoted with bold font.

Model Optimisation	Classifier	Accuracy/Day			
		1	7	21	All ^*b*^
GS	SVC	70.42±0.94	62.42±1.09	60.4±0.63	65.06±0.83
	LSVC *^a^*	72.89±0.33	67.79±0.64	62.23±0.85	68.08±0.52
	nu-SVM	69.69±0.42	58.73±0.55	56.93±0.33	62.66±0.39
	KNN	66.29±0.3	58.74±0.13	55.11±0.19	60.66±0.06
	MLP	67.64±0.49	62.01±0.94	59.69±0.52	63.57±0.60
GA	nu-SVM	73.27±0.55	63.42±0.75	60.54±0.48	66.54±0.45

^*a*^ SVM with a linear kernel; ^*b*^ results for combined data from all days.

**Table 6:** Results of the HICVS scenario for classification with GA and reference classifiers trained with a grid search (GS). The highest result in each day is denoted with bold font.

Model Optimisation	Classifier	Accuracy/Day			
		1	7	21	All ^*b*^
GS	SVC	71.29±0.65	64.16±1.21	62.85±0.99	66.67±0.88
	LSVC ^*a*^	72.79±0.44	68.4±0.81	62.8±1.12	68.38±0.66
	nu-SVM	71.75±0.95	63.86±1.69	63.67±1.24	67.04±1.16
	KNN	67.59±0.47	60.36±0.14	55.9±0.38	61.88±0.1
	MLP	67.49±0.44	62.14±1.02	59.68±0.83	63.54±0.66
GA	nu-SVM	75.17±1.91	73.42±2.11	69.99±2.54	73.02±2.14

^*a*^ Denotes SVM with a linear kernel; ^*b*^ Results for combined data from all days.

## Data Availability

The dataset is publicly available at the following link: https://zenodo.org/record/3984905 [Access date: 1 October 2020].

## References

[1] Ghamisi P., Plaza J., Chen Y., Li J., Plaza A.J. (2017). Advanced spectral classifiers for hyperspectral images: A review. IEEE Geosci. Remote Sens. Mag..

[2] Bioucas-Dias J.M., Plaza A., Camps-Valls G., Scheunders P., Nasrabadi N.M., Chanussot J. (2013). Hyperspectral remote sensing data analysis and future challenges. IEEE Geosci. Remote Sens. Mag..

[3] Bioucas-Dias J.M., Plaza A., Dobigeon N., Parente M., Du Q., Gader P., Chanussot J. (2012). Hyperspectral unmixing overview: Geometrical, statistical, and sparse regression-based approaches. IEEE J. Sel. Top. Appl. Earth Obs. Remote Sens..

[4] Landgrebe D.A. (2005). Signal Theory Methods in Multispectral Remote Sensing.

[5] Romaszewski M., Głomb P., Cholewa M. (2016). Semi-supervised hyperspectral classification from a small number of training samples using a co-training approach. ISPRS J. Photogramm. Remote Sens..

[6] Vapnik V., Sterin A. (1977). On structural risk minimization or overall risk in a problem of pattern recognition. Autom. Remote Control.

[7] Manolakis D., Marden D., Shaw G.A. (2003). Hyperspectral image processing for automatic target detection applications. Linc. Lab. J..

[8] Holland J.H. (1992). Adaptation in Natural and Artificial Systems.

[9] Rutkowski L. (1992). Computational Intelligence: Methods and Techniques.

[10] Ma J.P., Zheng Z.B., Tong Q.X., Zheng L.F. An application of genetic algorithms on band selection for hyperspectral image classification. Proceedings of the 2003 International Conference on Machine Learning and Cybernetics (IEEE Cat. No. 03EX693).

[11] Sukawattanavijit C., Chen J., Zhang H. (2017). GA-SVM algorithm for improving land-cover classification using SAR and optical remote sensing data. IEEE Geosci. Remote Sens. Lett..

[12] Kumar R.K., Saichandana B., Srinivas K. (2016). Dimensionality reduction and classification of hyperspectral images using genetic algorithm. Indones. J. Electr. Eng. Comput. Sci..

[13] Zhuo L., Zheng J., Li X., Wang F., Ai B., Qian J. (2008). A genetic algorithm based wrapper feature selection method for classification of hyperspectral images using support vector machine. Geoinformatics 2008 and Joint Conference on GIS and Built Environment: Classification of Remote Sensing Images.

[14] Romaszewski M., Głomb P., Sochan A., Cholewa M. (2021). A dataset for evaluating blood detection in hyperspectral images. Forensic Sci. Int..

[15] Tadeusiewicz R. (2015). Automatic Understanding of Medical Images (Opening Lecture) The 2nd International Conference “Innovative Technologies in Biomedicine”.

[16] Kłeczek P., Lech M., Jaworek-Korjakowska J., Dyduch G., Tadeusiewicz R. (2018). Segmentation of black ink and melanin in skin histopathological images. Medical Imaging 2018: Digital Pathology.

[17] Kłeczek P., Dyduch G., Jaworek-Korjakowska J., Tadeusiewicz R. (2017). Automated epidermis segmentation in histopathological images of human skin stained with hematoxylin and eosin. Medical Imaging 2017: Digital Pathology.

[18] Jaworek-Korjakowska J., Kłeczek P., Tadeusiewicz R. (2017). Detection and classification of pigment network in dermoscopic color images as one of the 7-point checklist criteria. Recent Developments and Achievements in Biocybernetics and Biomedical Engineering, Proceedings of the 20th Polish Conference on Biocybernetics and Biomedical Engineering, Kraków, Poland, 20–22 September 2017.

[19] Lu G., Fei B. (2014). Medical hyperspectral imaging: A review. J. Biomed. Opt..

[20] Edelman G., Manti V., van Ruth S.M., van Leeuwen T., Aalders M. (2012). Identification and age estimation of blood stains on colored backgrounds by near infrared spectroscopy. Forensic Sci. Int..

[21] Melgani F., Bruzzone L. (2004). Classification of hyperspectral remote sensing images with support vector machines. IEEE Trans. Geosci. Remote Sens..

[22] Pal M., Maxwell A.E., Warner T.A. (2013). Kernel-based extreme learning machine for remote-sensing image classification. Remote Sens. Lett..

[23] Khodadadzadeh M., Li J., Plaza A., Bioucas-Dias J.M. (2014). A subspace-based multinomial logistic regression for hyperspectral image classification. IEEE Geosci. Remote Sens. Lett..

[24] Ghamisi P., Maggiori E., Li S., Souza R., Tarablaka Y., Moser G., De Giorgi A., Fang L., Chen Y., Chi M. (2018). New frontiers in spectral-spatial hyperspectral image classification: The latest advances based on mathematical morphology, Markov random fields, segmentation, sparse representation, and deep learning. IEEE Geosci. Remote Sens. Mag..

[25] Li S., Song W., Fang L., Chen Y., Ghamisi P., Benediktsson J.A. (2019). Deep learning for hyperspectral image classification: An overview. IEEE Trans. Geosci. Remote Sens..

[26] Fang B., Li Y., Zhang H., Chan J.C.W. (2018). Semi-supervised deep learning classification for hyperspectral image based on dual-strategy sample selection. Remote Sens..

[27] Engelbrecht A.P. (2007). Computational Intelligence: An Introduction.

[28] Tadeusiewicz R. (2015). Neural networks as a tool for modeling of biological systems. Bio-Algorithms Med-Syst..

[29] Back T., Hammel U., Schwefel H.P. (1997). Evolutionary computation: Comments on the history and current state. IEEE Trans. Evol. Comput..

[30] Nguyen H.T., Sugeno M. (1998). Fuzzy Systems, Modeling and Control.

[31] Sivanandam S., Deepa S. (2008). Introduction to Genetic Algorithms.

[32] Park H., Son D., Koo B., Jeong B. (2021). Waiting strategy for the vehicle routing problem with simultaneous pickup and delivery using genetic algorithm. Expert Syst. Appl..

[33] Zhou Y., Zhang W., Kang J., Zhang X., Wang X. (2021). A problem-specific non-dominated sorting genetic algorithm for supervised feature selection. Inf. Sci..

[34] D’Angelo G., Palmieri F. (2021). GGA: A modified genetic algorithm with gradient-based local search for solving constrained optimization problems. Inf. Sci..

[35] Alonso-Arévalo M.A., Cruz-Gutiérrez A., Ibarra R., García-Canseco E., Conte-Galván R. (2021). Robust heart sound segmentation based on spectral change detection and genetic algorithms. Biomed. Signal Process. Control.

[36] Dong X., Zhang H., Xu M., Shen F. (2021). Hybrid genetic algorithm with variable neighborhood search for multi-scale multiple bottleneck traveling salesmen problem. Future Gener. Comput. Syst..

[37] Pławiak P., Acharya U.R. (2020). Novel Deep Genetic Ensemble of Classifiers for Arrhythmia Detection Using ECG Signals. Neural Comput. Appl..

[38] Pławiak P. (2018). Novel Genetic Ensembles of Classifiers Applied to Myocardium Dysfunction Recognition Based on ECG Signals. Swarm Evol. Comput..

[39] Książek W., Abdar M., Acharya U.R., Pławiak P. (2019). A Novel Machine Learning Approach for Early Detection of Hepatocellular Carcinoma Patients. Cogn. Syst. Res..

[40] Pławiak P., Abdar M., Pławiak J., Makarenkov V., Acharya U.R. (2020). DGHNL: A New Deep Genetic Hierarchical Network of Learners for Prediction of Credit Scoring. Inf. Sci..

[41] Pedergnana M., Marpu P.R., Dalla Mura M., Benediktsson J.A., Bruzzone L. (2013). A novel technique for optimal feature selection in attribute profiles based on genetic algorithms. IEEE Trans. Geosci. Remote Sens..

[42] Nagasubramanian K., Jones S., Sarkar S., Singh A.K., Singh A., Ganapathysubramanian B. (2018). Hyperspectral band selection using genetic algorithm and support vector machines for early identification of charcoal rot disease in soybean stems. Plant Methods.

[43] Tsai F., Philpot W. (1998). Derivative analysis of hyperspectral data. Remote Sens. Environ..

[44] Majda A., Wietecha-Posłuszny R., Mendys A., Wójtowicz A., ydżba-Kopczyńska B. (2018). Hyperspectral imaging and multivariate analysis in the dried blood spots investigations. Appl. Phys. A.

[45] Scholkopf B., Smola A.J. (2018). Learning with Kernels: Support Vector Machines, Regularization, Optimization, and Beyond.

[46] Głomb P., Romaszewski M., Cholewa M., Domino K. (2018). Application of hyperspectral imaging and machine learning methods for the detection of gunshot residue patterns. Forensic Sci. Int..

[47] Schölkopf B., Smola A.J., Williamson R.C., Bartlett P.L. (2000). New support vector algorithms. Neural Comput..

[48] Zhang Z. (2016). Introduction to machine learning: K-nearest neighbors. Ann. Transl. Med..

[49] Ramchoun H., Amine M., Janati Idrissi M.A., Ghanou Y., Ettaouil M. (2016). Multilayer Perceptron: Architecture Optimization and Training. Int. J. Interact. Multimed. Artif. Intel..

[50] Grefenstette J. (1992). Genetic algorithms for changing environments. Ppsn.

[51] Glorot X., Bengio Y., Teh Y.W., Titterington M. (2010). Understanding the difficulty of training deep feedforward neural networks. Proceedings of the Thirteenth International Conference on Artificial Intelligence and Statistics.

[52] Pedregosa F., Varoquaux G., Gramfort A., Michel V., Thirion B., Grisel O., Blondel M., Prettenhofer P., Weiss R., Dubourg V. (2011). Scikit-learn: Machine Learning in Python. J. Mach. Learn. Res..

[53] Paszke A., Gross S., Massa F., Lerer A., Bradbury J., Chanan G., Killeen T., Lin Z., Gimelshein N., Antiga L., Wallach H., Larochelle H., Beygelzimer A., d Alché-Buc F., Fox E., Garnett R. (2019). PyTorch: An Imperative Style, High-Performance Deep Learning Library. Advances in Neural Information Processing Systems 32.

[54] Fortin F.A., De Rainville F.M., Gardner M.A., Parizeau M., Gagné C. (2012). DEAP: Evolutionary Algorithms Made Easy. J. Mach. Learn. Res..

[55] Kandaswamy C., Silva L.M., Alexandre L.A., Santos J.M., de Sá J.M. (2014). Improving Deep Neural Network Performance by Reusing Features Trained with Transductive Transference. Artificial Neural Networks and Machine Learning—ICANN 2014.

